# Development of a liquid chromatography high resolution mass spectrometry method for the quantitation of viral envelope glycoprotein in Ebola virus-like particle vaccine preparations

**DOI:** 10.1186/s12014-016-9119-8

**Published:** 2016-09-05

**Authors:** Lisa H. Cazares, Michael D. Ward, Ernst E. Brueggemann, Tara Kenny, Paul Demond, Christopher R. Mahone, Karen A. O. Martins, Jonathan E. Nuss, Trevor Glaros, Sina Bavari

**Affiliations:** 1 Molecular and Translational Sciences Division, U.S. Army Medical Research Institute of Infectious Diseases, Frederick, MD 21702 USA; 2 BioSciences Division, Biodefense Branch, Edgewood Chemical Biological Center, Gunpowder, MD 21010 USA; 3 DOD Biotechnology High Performance Computing Software Applications Institute, Telemedicine and Advanced Technology Research Center, US Army Medical Research and Materiel Command, Fort Detrick, MD 21702 USA; 4 Excet, Inc., 8001 Braddock Road, Suite 105, Springfield, VA 22151 USA

**Keywords:** Ebola virus, Virus like particles, High resolution mass spectrometry, Stable isotope dilution quantitation

## Abstract

**Background:**

Ebola virus like particles (EBOV VLPs, eVLPs), are produced by expressing the viral transmembrane glycoprotein (GP) and structural matrix protein VP40 in mammalian cells. When expressed, these proteins self-assemble and bud from ‘host’ cells displaying morphology similar to infectious virions. Several studies have shown that rodents and non-human primates vaccinated with eVLPs are protected from lethal EBOV challenge. The mucin-like domain of envelope glycoprotein GP_1_ serves as the major target for a productive humoral immune response. Therefore GP_1_ concentration is a critical quality attribute of EBOV vaccines and accurate measurement of the amount of GP_1_ present in eVLP lots is crucial to understanding variability in vaccine efficacy.

**Methods:**

After production, eVLPs are characterized by determining total protein concentration and by western blotting, which only provides semi-quantitative information for GP_1_. Therefore, a liquid chromatography high resolution mass spectrometry (LC-HRMS) approach for accurately measuring GP_1_ concentration in eVLPs was developed. The method employs an isotope dilution strategy using four target peptides from two regions of the GP_1_ protein. Purified recombinant GP_1_ was generated to serve as an assay standard. GP_1_ quantitation in 5 eVLP lots was performed on an LTQ-Orbitrap Elite and the final quantitation was derived by comparing the relative response of 200 fmol AQUA peptide standards to the analyte response at 4 ppm.

**Results:**

Conditions were optimized to ensure complete tryptic digestion of eVLP, however, persistent missed cleavages were observed in target peptides. Additionally, N-terminal truncated forms of the GP_1_ protein were observed in all eVLP lots, making peptide selection crucial. The LC-HRMS strategy resulted in quantitation of GP_1_ with a lower limit of quantitation of 1 fmol and an average percent coefficient of variation (CV) of 7.6 %. Unlike western blot values, the LC-HRMS quantitation of GP_1_ in 5 eVLP vaccine lots exhibited a strong linear relationship (positive correlation) with survival (after EBOV challenge) in mice.

**Conclusions:**

This method provides a means to rapidly determine eVLP batch quality based upon quantitation of antigenic GP_1_. By monitoring variability in GP_1_ content, the eVLP production process can be optimized, and the total amount of GP_1_ needed to confer protection accurately determined.

**Electronic supplementary material:**

The online version of this article (doi:10.1186/s12014-016-9119-8) contains supplementary material, which is available to authorized users.

## Background

Ebola is an extremely pathogenic virus that causes hemorrhagic fever and can result in mortality rates of up to 90 %. The 2014 Ebola outbreak in West Africa brought global attention to a disease that was once only an isolated-regional problem. More than a year later and with a death toll greater than 10,000, there is an urgent need for novel therapeutic strategies including treatment and prevention. Virus-like-particles (VLPs) represent a new type of prophylactic vaccine that has had success and is commercialized in products such as Cervarix (human papillomavirus) and Gardasil (human papillomavirus) [1, 2]. VLPs are generated by exploiting the intrinsic ability of structural viral proteins, frequently major proteins in the capsid or envelop, to spontaneously self-assemble when expressed in mammalian cells [3]. VLPs are therefore composed of a subset of viral components that mimic the wild-type virus structure but lack viral genetic material, rendering them non-infectious. Unlike recombinant protein vaccines which may elicit a weak immune response due to non-ideal presentation of the viral antigens to the immune system, VLPs are usually antigenically indistinguishable from infectious virus particles [4–6]. These properties make VLPs promising candidates for new efficacious vaccines against many viral pathogens including filoviruses such as Ebola.

Ebola Virus (EBOV) VLPs (eVLPs) are produced by transfection of HEK293 cells with plasmids encoding the genes for viral matrix protein VP40 and envelope glycoprotein (GP) [7–9]. The envelope GP is solely responsible for viral attachment, fusion, and entry of new host cells, and it is therefore a major target of vaccine design efforts. When these proteins are expressed in mammalian cells, they self-assemble and bud from lipid rafts resulting in eVLPs that contain GP, VP40, and other packaged host proteins [10].

**Fig. 1:**
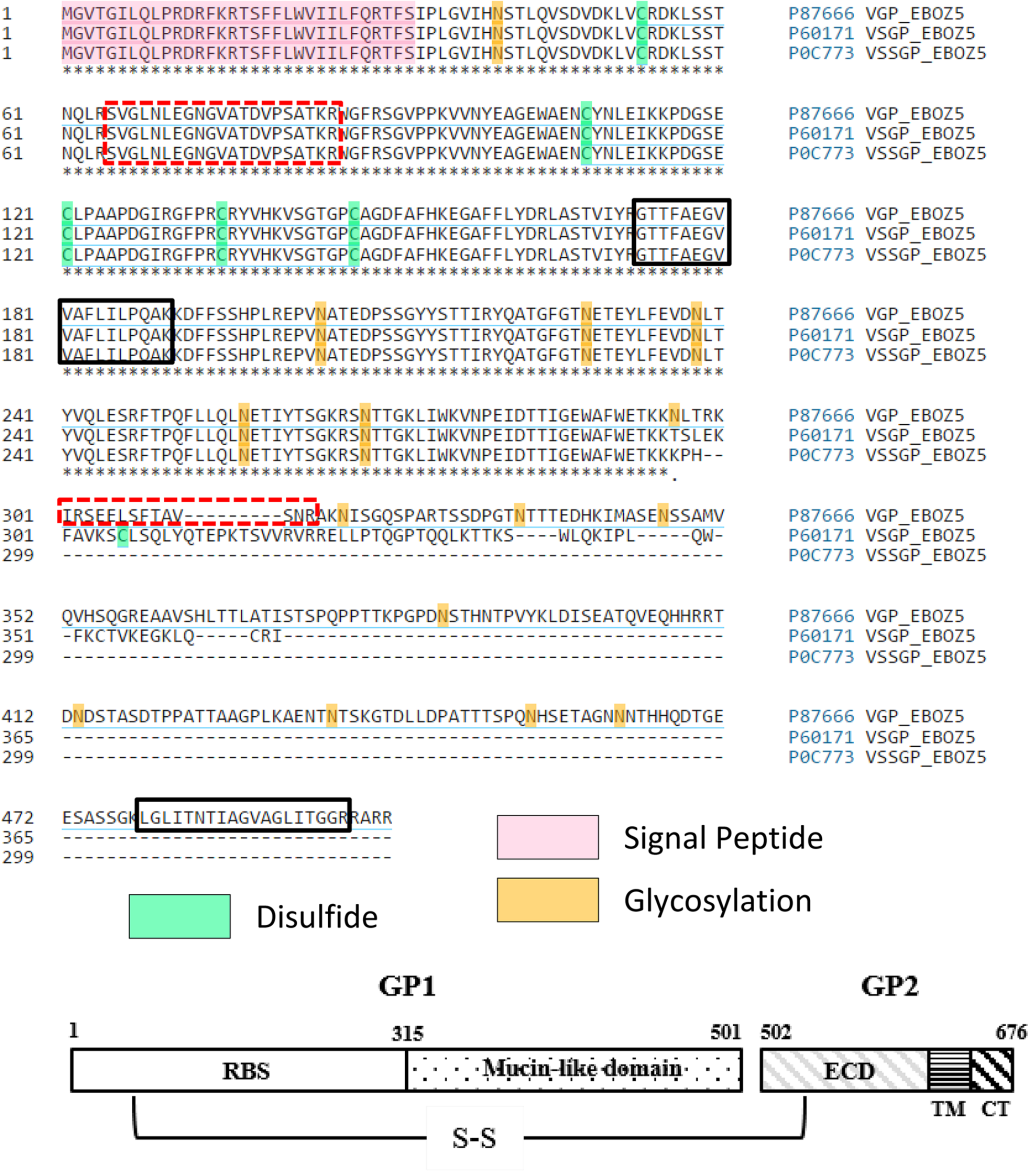
Target peptide selection and characterization. *Top* Sequence alignment of the 3 proteins (GP_1_, sGP and ssGP) derived from the Ebola GP transcript showing the locations of target peptide candidates for use in the quantification of Ebola GP_1_ (*red dotted boxes*) as well as the location of peptides rejected for the final assay (*black boxes*). All three protein products share sequence homology in the first 295 amino acids. Peptides identified in survey runs were evaluated for absence of post translational modifications, ionization efficiency and protein location. *Bottom* Schematic of fully processed GP_1,2_ transmembrane protein, showing the location of the receptor binding site (RBS) and mucin-like domain (MLD) of GP_1_, as well as the extracellular domain (ECD), transmembrane region (TM) and cytoplasmic tail (CT) of GP_2_. GP_1_ and GP_2_ are disulfide linked to form the mature GP_1,2_ complex

Each of the seven genes which comprise the EBOV genome is transcribed into individual messenger RNAs (mRNAs) with the exception of the fourth gene, which encodes for GP. In virus-infected cells, several GP-specific mRNAs are synthesized due to a transcriptional RNA editing phenomenon. Envelope GP is not the primary product of the fourth gene but instead is generated through transcriptional editing, which induces the EBOV polymerase to add an extra adenosine into a stretch of seven other adenosine residues at a specific-editing site near the middle of the coding region [11]. The EBOV polymerase transcribes the unedited GP gene which contains seven adenosines at the editing site most of the time (>80 %), and these transcripts result in the expression of the predominant GP gene product, secretory glycoprotein (sGP) [12]. The addition of 2 adenosine residues at the editing site (total of 9) codes for a third GP gene product known as second secreted GP (ssGP). Both secreted forms have the same amino-terminal 295 amino acids as envelope GP (see Fig. 1 for sequence alignment). Editing of the transcript (8 adenosines), results in the continuation of translation for an additional 381 amino acids beyond the editing site resulting in production of the pre-processed GP polypeptide (GP0). GP0 is cleaved into a large N-terminal portion (GP_1_) and a smaller C-terminal portion (GP_2_) in the *trans*-Golgi network by the subtilisin-like proprotein convertase, furin [13]. Mature envelope GP is formed by the re-joining of GP_1_ and GP_2_ through disulfide bonding, and the GP_1,2_ complex is anchored in the membrane by a transmembrane domain near the C-terminus of GP_2_ [14, 15]. GP_1_ contains a highly glycosylated mucin-like domain (MLD) and antibodies that recognize this region have been shown to be protective in mouse models of lethal Ebola virus challenge [16]. In addition, many neutralizing antibodies, including two that comprise part of a promising therapeutic cocktail [17], are directed against the MLD [16, 18, 19].

The GP expression vector used to produce eVLP in HEK293 cells encodes for a transcript containing 8 adenosines and thus should produce only GP_1,2_. Large scale production of eVLPs is performed by contract manufacturing organizations and each lot is characterized after production by assays that measure total protein and GP_1_ concentrations (western blotting or single antibody ELISA). Ongoing vaccine studies in our laboratory have shown that eVLPs provide protection against a lethal dose of EBOV in mice and non-human primates when administered with an appropriate adjuvant [20, 21]. Vaccine dosages are administered based on GP_1_ protein concentration; however, the effectiveness (based on survival) of each small scale VLP preparation can be highly variable. Therefore improved methods are needed to serve as lot release assays for each eVLP preparation to ensure that only material of sufficient quality is used for in vivo evaluation.

This report describes the development of an isotope dilution full scan LC-HRMS method for the absolute quantitation of Ebola GP_1_ in eVLP. The protocol resulted in the quantitation of GP_1_ with a lower limit of quantitation of 1fmol and an average percent coefficient of variation (%CV) of 7.6 %. The optimized MS quantitation of GP_1_, in contrast to the western blot quantitation, correlated with survival in vaccinated mice after EBOV challenge. This assay provides a means to monitor eVLP batch variability based on GP_1_ content, provides information for the optimization of production techniques, and will assist in the determination of the dosage needed to confer protection in vaccinated animals.

## Methods

### Generation and characterization of eVLPs

eVLPs were produced under a contract with Paragon Bioservices (Baltimore, MD) using a modification of the procedure described by Warfield et al. [22]. In brief, eVLPs were created by transfecting HEK 293 cells with expression vectors containing the genes for envelope GP and VP40 proteins [7, 22–24]. To purify the eVLPs, the clarified cell supernatants were pelleted, separated on a 20–60 % continuous sucrose gradient, concentrated by a second centrifugation, and resuspended in endotoxin-free PBS. The gradient fractions containing the eVLPs were determined via western blotting using an anti-GP_1_ antibody (6D8). The total protein concentration of each eVLP preparation was determined in the presence of Nonidet P-40 detergent using a detergent-compatible protein assay (Bio-Rad). For these blots unpurified recombinant GP material was used as an assay standard for the generation of a standard curve and quantitative information (performed by the contractor).

### Generation and characterization of a recombinant GP_1_ standard

A batch of recombinant Ebola glycoprotein (rGP, carrying an N-terminal poly-histidine tag) was expressed in human HEK293 cells and subsequently purified by immobilized metal affinity chromatography (IMAC). The material was produced under a contract with the Frederick National Laboratory for Cancer Research (Frederick, MD). Analytical scale reverse phase chromatography was used to further fractionate the protein preparation under reducing conditions. Recombinant Ebola glycoprotein material was reduced with 2-mercaptoethanol (final concentration, 0.5 M) during a 30 min room temperature incubation and then injected (300 µg total protein) onto an apHera C4 column (150 mm × 4.6 mm, 5 µm; Supelco). Mobile phases were as follows: (A) 0.1 % trifluoracetic acid (TFA) and (B) acetonitrile/0.1 % TFA. The flow rate was set to 0.5 mL/min and rGP was separated using the following gradient: 0–3 min: 10 % B, 3–5 min: 10–20 % B, 5–65 min: 20–45 % B, 65–71 min: 45–80 % B, and 72–82 min: 80–10 % B. During the 20–45 % B gradient, nine peaks were collected and dried to completion in a vacuum concentrator. All GP_1_ purification experiments were conducted using an Agilent 1200 HPLC system equipped with a UV detector; eluents were continuously monitored at 214 nm.

Each fraction of purified rGP_1_ was re-dissolved in 100 µL of 8 M urea/PBS. The protein concentration of each fraction was estimated by measuring the optical density (OD) at 280 nm in a spectrophotometer and assuming an extinction coefficient at 1 % equal to 10 (under this assumption, a 1 mg/mL solution of a protein would have an OD reading of 1.0). Protein from each fraction (500 ng) and 1 µg of the original unfractionated GP material were resolved on a 4–12 % BOLT SDS PAGE gel (Life Technologies) and stained with silver (Pierce Silver Stain kit, Fisher Scientific) following the manufacturer’s instructions. Following the initial characterization experiment, a larger scale purification experiment was conducted to obtain a sufficient quantity of GP_1_. In this iteration, 300 µg of unpurified recombinant GP material was fractionated by reverse phase HPLC and a single peak corresponding to GP_1_ was manually collected. The OD at 280 nm was recorded and a preliminary protein concentration was determined for the sample using a theoretical molar extinction coefficient of 54,768 (calculated from the primary sequence of GP_1_ using the protein parameter tool on the ExPASy server, http://web.expasy.org/protparam/). The sample was subsequently aliquoted and dried under vacuum centrifugation. SDS PAGE was used to compare the rGP_1_ pool to the original unfractionated rGP material. For this experiment, 2.5 µg of rGP_1_ and 3.3 µg of unfractionated rGP were resolved on a 4–12 % BOLT SDS PAGE gel (Life Technologies) and stained with Coomassie Blue (Imperial protein stain, Fisher Scientific). Lastly, the protein content of the pooled and purified rGP_1_ preparation was determined by amino acid analysis (AAA) following acid catalyzed hydrolysis by Biosynthesis (Lewisville, TX). AAA conducted on triplicate rGP_1_ samples determined that on average, each aliquot contains 1.8 µg of protein.

### Western blot analysis

Based on total protein concentration, approximately 20–50 ng of each eVLP lot was loaded onto a 4–12 % SDS PAGE gel and run under reducing conditions. Known amounts of recombinant Ebola GP material (purified GP_1_ and unpurified) were also loaded on the gel. Two separate gels were run for the eVLP lots tested and transferred to PVDF membranes. Each blot was blocked overnight with Odyssey blocking buffer in phosphate buffered saline (PBS) (LI-COR Biosciences Lincoln, NE) and then incubated with primary antibody against GP_1_ (6D8 or F88.H3D5, 1:1000) for 1 h at room temperature. After washing 3× with PBS + 0.1 % Tween-20 for 5 min, secondary antibody (1:5000) goat α-mouse IRDye^®^ 680 labelled (LI-COR) was added and the blots were incubated an additional hour. The blots were again washed 3× with PBST, and then stored in PBS until visualized with an Odyssey infrared imaging system (LI-COR Biosciences Lincoln, NE: model number 9210).

### Preparation of eVLP and rGP_1_ standard proteolytic digests

Upon receipt of each lot of eVLP from the contractor, stocks were divided into 10 µg aliquots based on the total protein concentration and stored at −80 °C until use. For simplicity, each of the 5 lots of eVLP used in this study was designated using alphabetical values (A–E). Sample preparation for MS was performed by first increasing the volume of each aliquot to 50µL with ‘Solution tA**’** (25 mM Tris–HCl, pH 8.0), reducing with 55 mM DTT at 55 °C for 30 min, and then alkylating with 68 mM iodoacetamide at room temperature for 45 min. Both of these steps were performed in the presence of 0.05 % ProteaseMax™ (Promega Madison, WI). The total volume was then increased to 95 µL with ‘Solution tD’ (25 mM Tris–HCl, pH 8.0, 10 % acetonitrile) and 4 µL of a 0.1 µg/µL sequencing grade trypsin/lys-C solution (Promega) and 1 µL of 1 % ProteaseMax™ were added followed by incubation at 42 °C for 4 h. Digests were heated to 90 °C for 5 min, dried completely by speed-vac and stored at −80 °C until analyzed. The purified rGP_1_ standard was digested using the same protocol as the eVLPs with the exception that the concentration of the trypsin/lys-C was reduced fourfold.

### Quantitation of GP_1_ by LC-HRMS

AQUA Ultimate™ peptides (Thermo Fisher Scientific) were synthesized based on the results of extensive survey runs of purified and digested rGP_1_ to determine which endogenous peptide sequences had the fewest possible post-translational or artefactual modifications and resulted in unambiguous MS^2^ spectra for identification, as well as consistent and chromatographically distinct extracted ion chromatograms (XIC) for quantitative measurement. The following four peptide sequences were selected: *301*-*IRSEELSFTAVSNR*-*314*, *303*-*SEELSFTAVSNR*-*314*, 65-*SVGLNLEGNGVATDVPSATK*-84, and 65-*SVGLNLEGNGVATDVPSATKR*-85. Each peptide had a C-terminal amino acid modified with ^13^C and ^15^N isotopes resulting in a 10 and 8 Da mass increase for arginine and lysine respectively. AQUA peptides were supplied by the manufacturer in a 5 % acetonitrile, 0.1 % formic acid solution at 5 pmol/µL. A 2× working solution was prepared in 40 % acetonitrile, 0.1 % formic acid by adding 8 µL of each stock peptide into a total volume of 200 µL (200 fmol/µL). The analyte digest was resuspended in 60 or 80 µL 40 % acetonitrile, 0.1 % formic and a 4-point, twofold serial dilution performed. AQUA peptides were then spiked into each analyte dilution at a 1:1 (*v*:*v)* ratio resulting in a 100 fmol/µL AQUA standard concentration. In addition, a blank was prepared by diluting the AQUA standards 1:1 with 40 % acetonitrile, 0.1 % formic acid. Samples were resolved on an Acclaim PepMap 100 column (1 mm × 100 mm) packed with 3um, 100A C18 particles and analyzed in triplicate from lowest to highest concentration by loading 2 µL onto an Ultimate 3000 HPLC (Thermo Fisher Scientific). Mobile phases were as follows: (A) 0.1 % formic acid (FA) and (B) acetonitrile/0.1 % FA. The flow rate was set to 75 µL/min and peptides were eluted using a 17-min linear gradient of 1–34 % mobile phase B. The column eluent was connected to an Orbitrap Elite mass spectrometer with a HESI-2 ion source (Thermo Fisher Scientific) using a sheath gas pressure of 20 psi and an auxiliary gas flow of 5 units. The electrospray ionization voltage was 5.0 kV with an ion transfer tube temperature of 350 °C and S-lens RF at 50 %. The automatic gain control target was 5.0 × 10^4^ for Orbitrap in SIM mode and 1.0 × 10^4^ for linear ion trap in MS/MS mode. The maximum injection time for MS/MS was set to 30 ms. Four consecutive 200 amu SIM scans over the range of m/z 415–1215 at a resolution of 60,000 were used to detect the ions of interest followed by 4 targeted MS/MS low resolution CID scans of the most prominent analyte peptides for sequence verification. For each peptide (heavy and light), both the doubly and triply charged ions were considered and used for quantitation. The average of triplicate extracted ion chromatogram (XIC) counts of each of the 4 standard AQUA peptides, the 4 analyte peptides and deamidated SVG peptides were obtained using XCalibur 2.0 (Thermo Scientific) with automatic integration baseline window set at 10 scans, area noise factor at 5, and peak noise factor set to 20. The XIC counts from each SVG, SVGR, SVG^deam^, and SVGR^deam^ peptide charge state were first summed in each individual replicate run and then the average for the three technical repeats was determined to represent the contribution of peptide Set 2 at each dilution. The SEE and IRSEE (peptide Set 1) values were obtained similarly. The AQUA standard peptide XIC counts were then used to calculate the ratio of AQUA peptide standard to the ‘light’ analyte peptide at each dilution using a mass tolerance of 4 ppm. This ratio or relative response was used to generate standard curves which were then used to determine the amount of analyte in fmols injected on-column. These fmol values were then converted to µg to calculate the total GP_1_ using a total protein mass of 50,916 Da (UniProt entry Q05320, 33-501).

### Limit of quantitation and linearity of analyte peptides

A previously quantified digest of a eVLP lot ‘A’ was diluted to 140 fmol/µL GP_1_ in 40 % acetonitrile, 0.1 % formic acid and serially diluted twofold down to 0.5 fmol/µL for a total of 9 dilutions. Using a 2 µL injection volume, each dilution was run in triplicate as described above and XIC area standard curves generated for each of the 4 quantitation peptides ranging from 275 to 1.0 fmol. The similar procedure was carried out on the AQUA peptides except the dilution was carried to 0.4 fmol/µL.

### Deamidation of AQUA peptide standards

A 40 pmol aliquot of AQUA SVG peptide was resuspended in 200 µL 50 mM NH_2_HCO_3_ pH 8.1 and incubated at 50 °C for 3 days then dried to completion by speed-vac. The sample was resuspended in 200 µL 40 % acetonitrile, 0.1 % formic acid and 2 µL was injected using the instrument and chromatographic conditions outlined above. Target masses were aligned by charge state and retention time and XIC values were derived as described above using a mass tolerance of 4 ppm.

### In-gel trypsin digestion

A 5 µg aliquot of VLP was fractionated by SDS-PAGE under reducing conditions onto a 4–12 % gel (BioRad) and the 10 highest intensity bands excised and minced into 1 × 1 mm plugs. Each sample was serially processed in 100 µL solution tA, then solution tB (25 mM Tris–HCl, pH 8.0, 50 % Acetonitrile), and finally 100 % Acetonitrile before being evaporated to dryness in a vacuum concentrator. Each gel slice was then reduced and alkylated by incubation in 55 mM DTT at 55 °C followed by incubation with 68 mM iodoacetamide for 45 min at room temperature. Bands were dried to completion and 10 µL of a 12.5 ng/µL sequencing grade modified trypsin solution (Promega, Madison, WI) in solution tD was added and incubated at room temperature for 30 min until trypsin was absorbed. 70 µL solution tD was then added and samples incubated overnight at 37 °C. Peptides were then extracted 2× by incubating in 50 % Acetonitrile, 0.1 % formic acid and the combined digest were dried to completion in a vacuum concentrator.

### Animals, vaccinations, and viral challenge

Research was conducted under an IACUC approved protocol in compliance with the Animal Welfare Act, PHS Policy, and other Federal statutes and regulations relating to animals and experiments involving animals. The facility where this research was conducted is accredited by the Association for Assessment and Accreditation of Laboratory Animal Care, and adheres to principles stated in the Guide for the Care and Use of Laboratory Animals, National Research Council, 2011. C57BL/6 mice were obtained from NCI Charles River. Female mice between 8 and 12 weeks of age were vaccinated with 100 µL injections containing 10 µg of GP (as determined by western blot) via the intramuscular (IM) route, in the caudal thigh. Each lot of eVLP was irradiated at 1e6 rad to ensure sterility and contained less than 25 EU/mL endotoxin and less than 10 colony forming units (CFU) of bacteria per vaccination. VLP were diluted in sterile saline and vaccinations were administered two times, with 3 weeks between vaccinations. Viral challenge occurred 4 weeks after the second vaccination. A challenge dose of 1000 pfu of mouse-adapted Ebola virus [25] was administered via the intraperitoneal route (IP). The survival data was pooled from tow studies with n = 10 mice each.

### Statistical analysis (differences between lots, animal survival rates)

Survival studies were evaluated using Fisher’s exact test with multiple testing corrections performed by permutation based on the number of comparison’s performed. The significance of the deviation from a null hypothesis (p value) was reported for the survival observed in animals vaccinated with each eVLP lot.

## Results

### Selection and evaluation of GP_1_ target peptides for quantitation by LC-HRMS

**Fig. 2:**
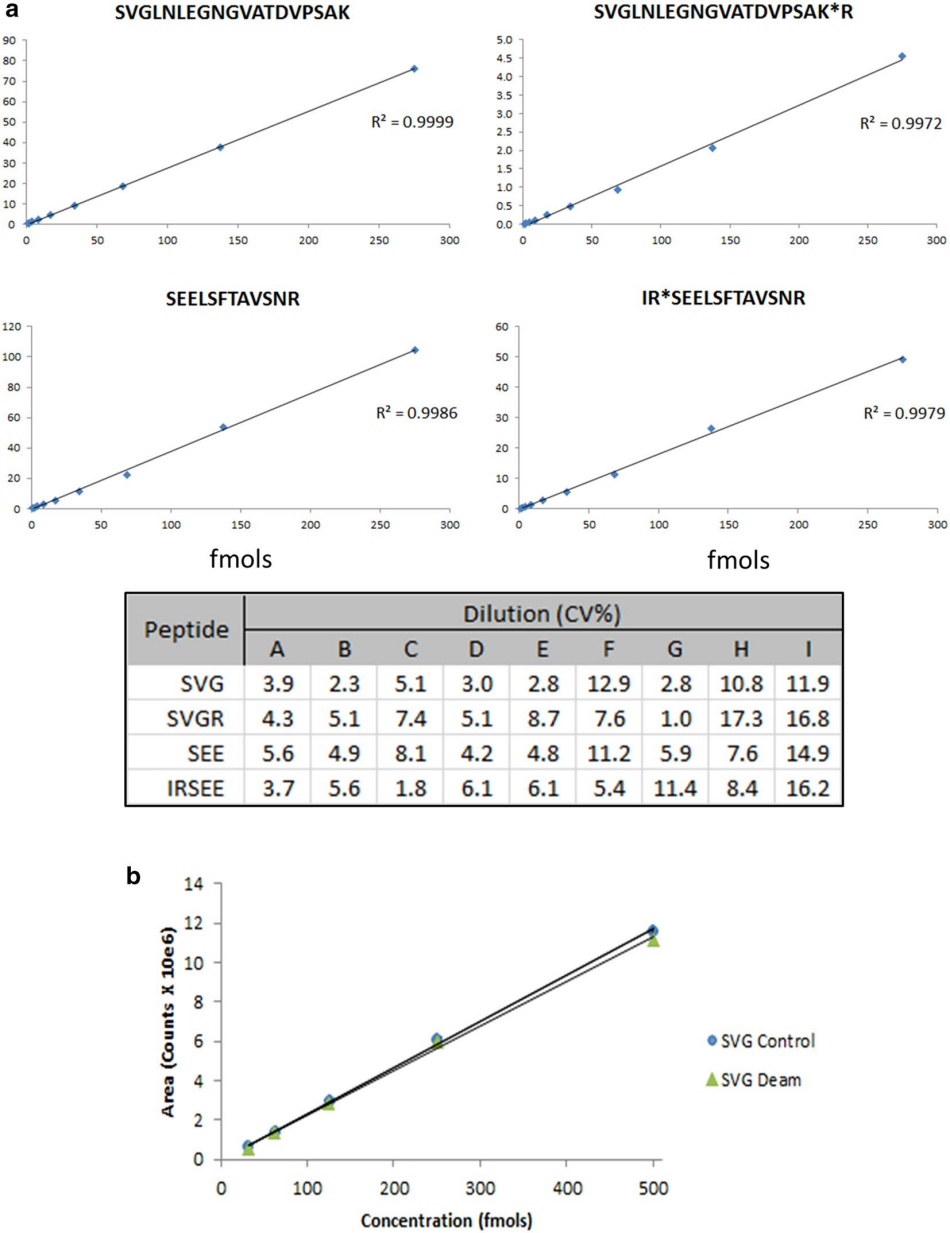
Characterization of target peptide standards. **a** Standard curves for each target analyte peptide over a 9 point dilution showing linearity from 275 fmols to 1 fmol total GP_1_. An aliquot of the previously quantified eVLP lot ‘A’ (200 fmols/µL SEE at 120 µL dilution) was resuspended 83 µL 40 % acetonitrile, 0.1 % Formic (137.5 fmols/µL) and serially diluted. A 2 µL injection utilizing the described instrument method was run in triplicate for each dilution. R^2^ values for all four peptides are well within the margin of significance for linearity. Also shown in tabular form are the %CV values for each triplicate XIC measurement for each peptide at each dilution. These data indicate linearity down to 1 fmol with the largest CV% (SVGR—17.3 %) in dilution number ‘8’ of the serially diluted series. **b** AQUA-SVG peptide signal response for non-deamidated (*circle*) and deamidated (*triangle*) peptide. AQUA-SVG peptide was deamidated by incubating 40 pmols at 50 °C/pH 8.0 for 2.5 days while a matching 40 pmol aliquot was stored at −20 °C. A 5-point, twofold serial dilution was performed resulting in a 250–15.6 fmol/µL concentration range for each sample. LC-HRMS was run in triplicate on each dilution and the average counts plotted

In the development of a reproducible MS protein quantitation scheme, the selection of target peptides is a crucial step, especially when the protein of interest is expressed in multiple isoforms, and is highly post-translationally modified. In both the infectious virions and eVLP preparations, GP_1_ and GP_2_ are proteolytically processed from the GP0 polypeptide and disulfide linked to form the mature GP_1,2_ transmembrane protein complex [2, 15] (see Fig. 1). Four peptides were initially identified as target candidates for the quantitation of GP_1_ primarily due to their ionization characteristics, lack of post-translational modifications and relative distance within the sequence. During initial LC-HRMS method development it was discovered that two of these peptides (173-*GTTFAEGVVAFLILPQAK[*^*13*^*C6,*^*15*^*N2]*-190) and (479-*LGLITNTIAGVAGLITGGR[*^*13*^*C6,*^*15*^*N4]*-497) failed to show consistent linearity. The GTT peptide and LGL peptide have a Grand Average of Hydropathy score (GRAVY) [26] of 0.933 and 1.08 respectively, indicating a high level of hydrophobicity, which can hinder reliable quantitation. The remaining 2 peptides 65-*SVGLNLEGNGVATDVPSATK[*^*13*^*C6,*^*15*^*N2]*-84 and 303-*SEELSFTAVSNR[*^*13*^*C6,*^*15*^*N4]*-314 (designated SVG and SEE, respectively) provided highly reproducible linear standard curves and were selected for use in the assay (see Figs. 1, 2a). The selection of these 2 peptides also offered a way to distinguish envelope GP_1_ from amino-terminal sequences containing fragments of the protein, as the SEE peptide sequence is found only in the full length GP_1_ molecule. Isotopically labelled AQUA Ultimate™ peptides (Thermo Scientific) were synthesized for each target peptide sequence. Synthetic AQUA (Absolute QUAntitation) peptides are chemically and physically indistinguishable from their endogenous counterparts with respect to retention time, ionization efficiency, and MS/MS fragmentation except they are modified to include ^13^C and ^15^N isotopes that increase their relative mass by very precise increments [21]. For this study, AQUA Ultimate™ peptides were selected as they have the highest available concentration precision and purity.

 During the initial survey runs which were conducted to optimize digestion of the eVLP for completeness and reproducibility, it was observed that two missed cleavage sites appeared regularly: a C-terminal arginine on the SVG peptide and an N-terminal arginine on the SEE peptide. We rigorously searched for additional missed cleavages as well as non-specific cleavages upstream and downstream of the fully tryptic peptides, and found no evidence that these species were present (see Additional file 1: Table S1). Given that the ratio of missed cleavage to fully tryptic peptides was highly variable (0.4–45 %), the 2 peptides representing these missed cleavages (65-*SVGLNLEGNGVATDVPSATK****R****[*^*13*^*C6,*^*15*^*N4]*-85 and 301-***IR****SEELSFTAVSNR[*^*13*^*C6,*^*15*^*N4]*-314) were synthesized and evaluated for reproducibility and linearity. These peptides were chromatographically distinct, generated linear standard curves and were therefore suitable for use in the quantitation assay (see Fig. 2a; Additional file 2: Figure S1). It was also observed that one of the two asparagine residues within the endogenous SVG peptide, but not both, were routinely deamidated. Since all extracted-ion chromatogram (XIC) counts from this species must be combined with the non-deaminated values in order to account for the full stoichiometric contribution of the SVG peptide it was evaluated whether the non-deamidated AQUA SVG peptide standard could be used to quantitate the level of deamidated analyte peptide. The standard AQUA SVG and SVGR peptides were fully deamidated by incubating them at 55 °C for 2.5 days at pH 8.1, and evaluated using the developed LC-HRMS method. Interestingly, even with this harsh treatment, the doubly deamidated species comprised only 5 % of the total SVG peptide compliment, indicating that under normal processing conditions it would be a highly unlikely modification (see Additional file 3: Figure S2). The XIC response of the deamidated peptide standards were then compared to the non-treated peptide standard of the same concentration. As shown in Fig. 2b, the response was essentially identical. Therefore, the XIC counts derived from the SVG and SVGR standard AQUA peptides were used to quantify the additional XIC counts from the endogenous deamidated peptide species without necessitating the production of additional labeled deamidated standards. We did not observe deamidation of the single asparagine in the SEE target peptide.

### Determination of optimal digestion conditions for GP_1_ within the eVLPs

The proteolytic enzyme of choice is a mass spectrometry grade Trypsin/Lys-C combination (Promega #V5073) as it is well characterized, versatile and highly specific. Initial digestion experiments and LC-HRMS analysis of the eVLPs revealed that some regions of GP_1_ are very resistant to proteolytic digestion even in the presence of enhancing surfactants such as ProteaseMax™. To ensure complete digestion of the eVLP GP_1_, we conducted extensive testing using a variety of buffer formulations, reagents, and pre-digestion treatments. These treatments included deglycosylation, sonication and high temperature. Since GP_1,2_ is a heavily glycosylated membrane embedded protein, we performed PNGase deglycosylation prior to digestion in the hope of reducing steric hindrance of the sugars and thereby enhancing trypsin proteolysis. Although we observed a modest improvement in overall peptide count as well as a reduction in frequency of the SVG/SEE missed cleavages, we did not observe any appreciable differences in the ratios of the target peptides selected for use in quantitation (data not shown). Therefore it was concluded that the additional deglycosylation procedure would only add to the complexity of the assay. We also tested the cleavable detergent/surfactant, ProteaseMax™ (Promega, Madison, WI), which is designed to enhance the performance of trypsin, and is especially useful for membrane proteins. This reagent dramatically reduced the overall number of missed cleavages and allowed the digest time to be reduced from 16 to 4 h without any loss of digestion efficiency. Despite these efforts, we were unable to completely eliminate the occurrence of the target peptide missed cleavages described above. However, we did not observe any additional upstream or downstream missed cleavage species from either target peptide in survey runs from each eVLP lot tested (Additional file 1: Table S1). Missed cleavage species were observed in 8.2 % of the SEE peptide and 31 % in the SVG peptide. These values represent the typical level observed in all 5 eVLP lots tested after trypsin digestion. We therefore concluded that the 4 peptides selected for the assay would be adequate for quantitation of GP_1_ present in eVLP preparations. The final peptide sequences and charge states used for quantitation of Ebola GP_1_ are shown in Table 1.

**Table 1:** Masses of analyte and AQUA standard peptides used for quantitation of GP_1_ in eVLP

Set	Sequence	Analyte		AQUA standard	
		2(+) m/z	3(+) m/z	2(+) m/z	3(+) m/z
1	SEELSFTAVSNR	670.3281	447.2211	675.3322	450.5572
1	IRSEELSFTAVSNR	804.9206	536.9495	809.9248	540.2856
2	SVGLNLEGNGVATDVPSATK	964.9998	643.669	969.0069	646.3404
2	SVGLNLEGNGVATDVPSATKR	1043.0498	695.7027	1048.0549	699.0388
2	SVGLNLEGNGVATDVPSATK-deam	965.4918	643.997	N/A	N/A
2	SVGLNLEGNGVATDVPSATKR-deam	1043.5424	696.0307	N/A	N/A

### Reverse phase purification of GP_1_ standard

**Fig. 3:**
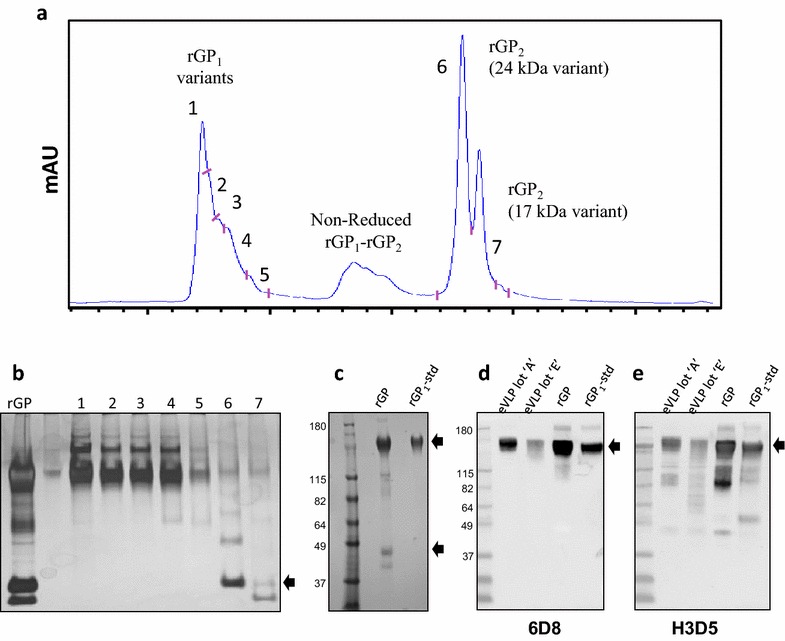
Purification and characterization of a recombinant GP_1_ standard. **a** Representative chromatogram of preparative C4 reverse phase HPLC of 300 µg reduced recombinant GP material indicating fraction collection points. **b** SDS PAGE followed by silver-staining of fractions 1–7 showing the separation of GP_1_ (*top arrow*) and GP_2_ (*bottom arrow*). Material from fraction 1 was divided into 1.8 µg aliquots and used for quantitation standard. **c** Silver stained SDS PAGE performed under reducing conditions comparing the rGP starting material and the purified rGP_1_ standard. (*top arrow*) GP_1_ and (*bottom arrow*) GP_2_. D) Western blot of eVLP Lot ‘A’, eVLP Lot ‘E’, unpurified rGP and the purified rGP_1_ standard using the monoclonal antibody 6D8 showing the detection of fully glycosylated GP_1_ (*arrow*). E) Western blot of eVLP Lot ‘A’, eVLP Lot ‘E’, unpurified rGP and the purified rGP_1_ standard using the monoclonal antibody H3D5 showing the detection of fully glycosylated GP_1_ (*arrow*) and GP protein fragments

In any protein quantitation experiment, the assumption is that unique peptides from different regions within a protein will display a 1:1 molar relationship. However, early quantitation experiments with test lots of recombinant GP material and eVLP revealed a variable target peptide (SVG:SEE) stoichiometric ratio (designated as ΔS below) between the lots which was otherwise consistent within each lot. In some cases the disparity between the SVG quantitation and the SEE quantitation was as high as 25 %. In order to rule out experimental error as the cause of the discrepancy, we prepared a pure monomeric full length rGP_1_ standard from recombinant GP material (containing GP_1_ and GP_2_) that could be used to assess the accuracy of the quantitation method. As shown in Fig. 3a, a reverse phase chromatography procedure was performed that fractionated reduced rGP material into multiple sub-species. Fractions were collected and evaluated by SDS PAGE analysis and silver staining. As seen in Fig. 3b, fractions 1–4 and fractions 6–7 constitute GP_1_ and GP_2_, respectively. Interestingly, fractions 1–4 yielded nearly identical SDS PAGE profiles despite observing multiple shoulder peaks on the reverse phase chromatogram. Ultimately however, the fractionation procedure resulted in a significant enrichment of individual protein species within the rGP preparation, and SDS PAGE analysis confirmed that the fractionated material was highly enriched for GP_1_ (see Fig. 3c). Collectively, this data indicates that the procedure significantly reduced the amount of heterogeneity in the original sample and produced an enriched version of GP_1_ that was suitable for use as an assay standard.

### Validation of the quantitation method with purified rGP_1_ standard

Quantitative amino acid analysis (AAA) indicated each aliquot of purified rGP_1_ contained an average of 1.8 µg GP_1_ protein. In order to evaluate the accuracy and precision of the assay, four rGP_1_ aliquots were resuspended in 80 µL 40 % acetonitrile with 0.1 % formic acid and quantitated in triplicate using the LC-HRMS assay. As seen in Table 2, after averaging the individual peptide set values, the GP_1_ concentration was determined to be 1.45 µg/aliquot for trial 1 and 1.52 µg/aliquot for trial 2. These values are within 20.5 and 15.5 % of the value obtained with AAA (1.8 µg). The SVG/SEE stoichiometric disparity (designated as ΔS), was 4.6 % for trial 1 and 5.5 % for trial 2 and the  %CV was 3.2. These data indicate that the LC-HRMS method using the combination of these 4 peptides (Set 1 and Set 2) was sufficient to account for the GP_1_ protein present with an average accuracy 82.5 %.

**Table 2:** HR/AM-MS method validation using purified recombinant GP_1_ standard

Trial	Dilution	Peptide	On-column		Total In sample (µg)	Ave. (µg)	ΔS (%)	Accuracy (%)	Precision (% CV)
			fmoles	ng					
1	80	Set 1	347.7	17.7	1.42	1.45	4.60	80.5	3.2
		Set 2	363.7	18.5	1.43				
2	80	Set 1	363.3	18.5	1.48	1.52	5.50	84.5	
		Set 2	383.4	19.5	1.56				

### Development of a high resolution/accurate mass (HR/AM) quantification of GP_1_ in eVLPs

Since the purified rGP_1_ standard returned acceptable LC-HRMS quantitation results, we sought to determine the source of the disparity observed in the quantitation of GP_1_ in the eVLPs when using peptide Set 1 and peptide Set 2 (ΔS). While the eVLPs are designed to produce only GP_1,2_ by altering the primary sequence used to transfect the HEK293 cells, the presence of multiple forms of GP was observed by western blotting using two monoclonal antibodies with epitopes located in different regions of the molecule (see Fig. 3d, e). The mouse monoclonal antibody 6D8 binds at amino acids 389-405 and therefore has affinity for Ebola GP_1_ only [16]. This is the antibody routinely employed for the determination of GP content in the eVLP preparations by quantitative western blot or ELISA. Antibody H3D5 is a mouse monoclonal antibody which binds at amino acids 72-109 and therefore has affinity for all forms of GP (both secreted and membrane bound) (see Fig. 1) each containing the SVG peptide sequence. This antibody has reactivity with all subtypes of Ebola GP_1_, for all subspecies [27]. As shown in Fig. 3d, e, the predominant band visualized using both antibodies in the unfractionated rGP material, purified rGP_1_, and two lots of eVLPs (lots ‘A’ and ‘E’), is fully glycosylated GP_1_ (~ 140 kDa). However the H3D5 blot shows the presence of strong distinct bands of a lower molecular weight (~50 to 100 kDa) present in both eVLP lots and the unpurified rGP material. These bands are much reduced in the rGP_1_ purified standard compared to the unpurified rGP material. The additional bands visible in the eVLP western blot using the H3D5 antibody do not correspond to the correct molecular weight for either sGP or ssGP (50 and 47 kDa respectively). In order to verify sequence identity these bands were excised from a gel of one eVLP lot (‘A’) and stained for total protein with coomassie blue. The 10 most intense bands were excised; trypsin digested, and analyzed with long-gradient CID survey runs as well as targeted LC-HRMS MS to identify any GP protein fragments contributing to the peptide quantitative variability. The results of this sequencing experiment are shown in Additional file 4: Figure S3. All bands excised were confirmed to contain EBOV GP_1_ or GP_2_ peptides. A gradual loss of C-terminal peptide identifications for GP_1_ was observed as the smaller products visible in the gel were sequenced, suggesting the presence of truncated forms of GP_1_ in the eVLP. This data indicates that peptides derived from the first ~ 200 amino acids of GP_1_ would not be suitable candidates for quantitation of the protein. We therefore concluded that the SEE target peptide (Set 1) was the only reliable standard for the quantitation of GP_1_ in eVLP.

### Testing of the LC-HRMS quantitation method for reproducibility with an eVLP digest

**Fig. 4:**
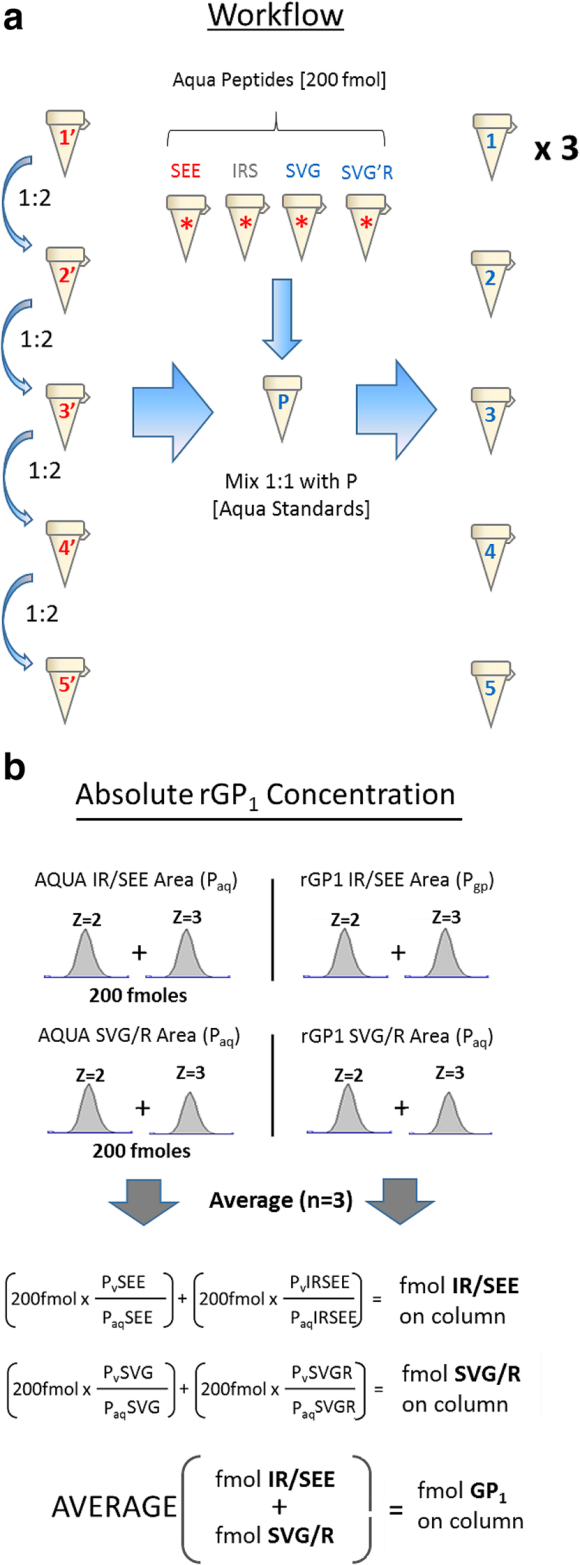
Illustration of the eVLP quantitation method workflow and calculations. **a** VLP digests are resuspended in 120 µL and four twofold serial dilutions performed. Each dilution is mixed 1:1 with a solution containing 200 fmol/µL of each of the four isotopically labeled AQUA peptides and run in triplicate using 2 µL injections. **b** Method used for calculating the GP_1_ concentration in the rGP_1_ standard at each dilution. Average XIC area counts from the 2+ and 3+ charge states are first summed for each AQUA (P_aq_) and analyte (P_v_) peptide. The values from the SEE and IRSEE are summed to provide the total counts for peptide ‘Set 1’ and the SVG and SVGR values are summed to provide total counts for peptide ‘Set 2’. The final quantitation is derived by comparing the relative response of the 200 fmol AQUA standard to the endogenous analyte response at 4 ppm and averaging the response between the 2 peptide sets. For absolute eVLP rGP_1_ quantitation, only the values derived from peptide set 1 (SEE/IRSEE) were used

Three aliquots from a single test lot of eVLP (lot ‘A’) were used to evaluate the quantitation method for reproducibility. As shown in Fig. 4a, the workflow was as follows: heavy AQUA standard peptides were added at a fixed concentration of 200 fmols/injection while varying the concentration of the eVLP analyte digest (10 µg based on total protein concentration) over 4 twofold dilutions for a total of 5 dilutions. Each complete quantitation set was run in triplicate with an analyte resuspension volume of 120µL. The XIC area contributions from each charge state were summed to provide the total fmols for each peptide species (see Fig. 4b; Table 3). Replicates 1, 2, and 3 resulted in a calculated GP_1_ concentration (based on the SEE peptide standard set 1 only) of 0.57, 0.49 and 0.53 mg/mL, respectively for an average of 0.53 mg/mL ± 0.04 mg/mL and a percent CV of 7.6. Therefore, each aliquot of this eVLP contained an average of 1.13 µg of GP_1_, or 11.3 % of the total protein concentration of 10 µg. The average ΔS (SVG/SEE stoichiometric disparity) value was 17.3 %. These data suggest that the newly developed LC-HRMS method can quantitate the amount of GP1 in eVLP reproducibly.

**Table 3:** GP_1_ quantitation in three replicates of a single eVLP lot

Sample	Digest (µg)	fmoles		Aliquot (µg)	Concentration (mg/mL)	Pct. total protein	∆S (%)	CV%
		Pep Set 1	Pep Set 2					
Lot A (1)	10	199.1	231.1	1.22	0.57	12.2	16.1	7.60 %
Lot A (2)	10	172.3	207.1	1.05	0.49	10.5	20.2	
Lot A (3)	10	183.8	212.2	1.12	0.53	11.2	15.5	

**Fig. 5:**
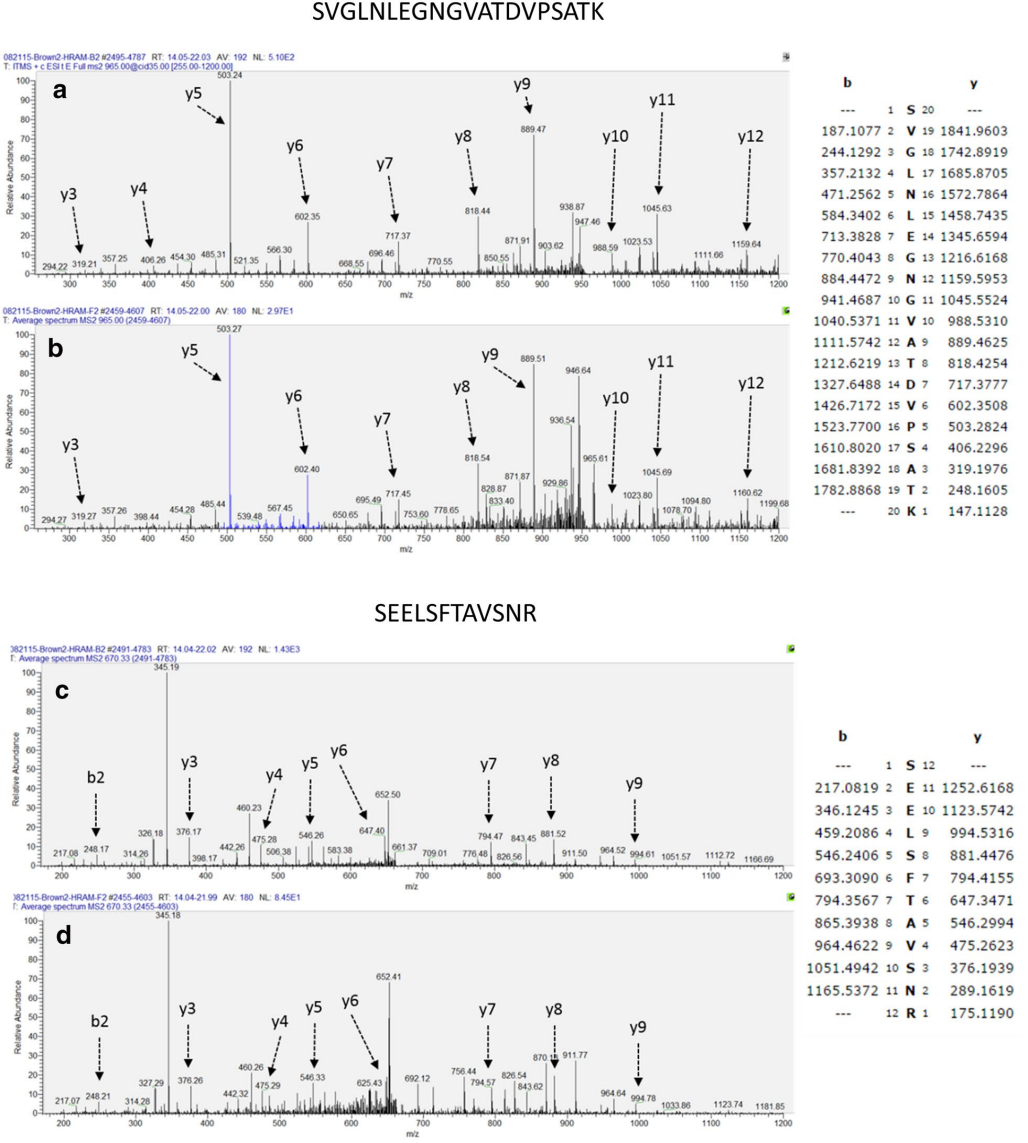
Average MS/MS fragmentation spectra of the SVG and SEE peptides. Replicate CID fragmentation spectra and 300 ppm theoretical ion tables of the SVG (**a**, **b**) and SEE (**c**, **d**) analyte peptides derived from eVLP lot ‘A’. **a**, **b** Represent the y-series assignments at the 1:2 (1) and 1:32 (5) dilution samples respectively (see dilution scheme in Fig. 4a). Prominent y-series sequence ions are indicated. The SVG series contains 10 consecutive y-series ions resulting in a MASCOT Ions Score of 69 and an Expect score of 6.7 × 10^−6^ at dilution ‘1’ with the ‘5’ dilution Ions Score at 57 with an Expect Score of 8.8 × 10^−5^. The SEE peptide contains 7 y-series ions in both the ‘1’ and ‘5’ dilutions with Ions Scores of 55 and 41 with Expect Scores of 8.2 × 10^−5^ and 0.0021 respectively. With the exception of the SEE ‘5’ dilution, peptide assignments from every dilution run were of sufficient quality to obtain non-ambiguous sequence identifications

In order to reduce the possibility of including peptide ion counts from contaminating ion species, our LC-HRMS method included a second stage MS/MS step to fragment each of the analyte peptides to confirm target sequence identity. We were able to confidently identify each of the 4 analyte quantitation peptides in at least the 3 highest dilutions of the test eVLP lot used to determine assay reproducibility. Representative MS/MS spectra of the SVG and SEE target analyte peptides are shown in Fig. 5. With the exception of the SEE peptide at the highest dilution, peptide assignments from every dilution run were of sufficient quality to obtain non-ambiguous sequence identifications.

### Linearity and limit of quantitation of analyte peptides

To assess the lower limit of quantitation of the assay, and to ensure the range of protein concentrations tested remains linear relative to our single standard peptide concentration, we performed a linearity and limit of quantitation experiment. While the observed range of concentrations over 5 dilution points spanned from 6 to 250 fmols, it was necessary to show that we could extrapolate to concentrations that fell outside the fixed concentration represented by the AQUA peptide standards. We therefore prepared a dilution of a previously quantified eVLP lot (‘A’) such that a 9-point twofold serial dilution resulted in an on-column GP_1_ load of between 275 fmol and 1 fmol. The averaged triplicate XIC values were plotted and %CV values determined (see Fig. 2a: tabular data). The quantitation remained linear across the entire concentration range (1–275fmol) with R^2^ values for SVG (0.9999), SVGR (0.9972), SEE (0.9986) and IRSEE (0.9979) well within the margin of linear significance. While %CV values at the highest dilutions were typically less than 5, the values in the 2 most dilute concentrations spanned a range of 7.6–17.3 %. This is within acceptable limits of variability and therefore the quantitative accuracy of the assay is reliable down to 1fmol.

### Quantitation of multiple lots of eVLP and comparison with western blot quantitation

The optimized protocol developed for the digestion and LC-HRMS quantitation of GP_1_ was performed on digests of 5 different lots of eVLPs. These lots were produced and characterized using western blot by an outside contractor (Paragon BioServices, Baltimore, MD) and, at the time of our study, were being used for a number of in-house animal vaccination studies. Using primary aliquots which were stored at −80 and would therefore experience only one freeze-thaw, eVLPs (10 ug total protein) were digested and triplicate LC-HRMS quantitation runs were performed. Resuspension volumes for all eVLP digests were 120 µL. As shown in Fig. 4b, the final quantitation was derived by comparing the relative response of the 200 fmol AQUA standards (SEE and IRSEE: Set 1) to the endogenous analyte response at 4 ppm at each dilution. The average XIC’s were then calculated and used to obtain the final concentration of GP_1_ protein present in the eVLPs. This quantitation was performed in duplicate and the concentrations of GP_1_ for all 5 lots of eVLPs are shown in Table 4. The lowest percentage of GP_1_ relative to the total protein concentration was found in lot ‘E’ (1.2 %) with a final concentration of 0.09 mg/mL GP_1_. The next lowest values are found in the lot ‘D’ (3.0 %, 0.15 mg/mL) while the highest percentage of GP_1_ relative to the total protein concentration was found in lot ‘A’ (11.7 %, 0.55 mg/mL). This represents nearly an order of magnitude difference in relative GP_1_ concentration between the VLP lots ‘A’ and ‘E’. The ΔS values for each eVLP lot tested ranged from 7.35 % for the lot ‘B’ to 25.5 % for lot ‘E’.

**Table 4:** LC-HRMS results of GP_1_ quantitation in 5 lots of eVLP and comparison with quantitative western blot values

Lot	Concentration (mg/mL)			MS (CV%)	Average ∆S (%)	GP1 %TP
	Protein	rGP WB	MS			
A	4.7	1.0	0.55	6.0 %	15.8	11.7
B	3.3	1.14	0.33	9.9 %	7.35	8.6
C	4.8	1.4	0.29	11.6 %	12.7	6.1
D	4.9	0.7	0.15	7.2 %	24.1	3.0
E	7.2	1.1	0.09	5.2 %	25.5	1.2

For each eVLP lot, the GP_1_ concentration was determined after production by the contractor via western blot with the 6D8 antibody and unpurified rGP material as a quantitation standard. As shown in Table 4, the range of concentration for GP_1_ was 0.71–1.4 mg/mL as determined by western blot. Total protein values provided for each lot ranged from 3.8 to 7.2 mg/mL. Since the western blot quantitation and the MS quantitation results were vastly different we investigated the source of this discrepancy by repeating the western blot on the eVLP lots with the highest and lowest calculated GP_1_ (as determined by LC-HRMS) using the 6D8 and H3D5 antibodies (see Fig. 3d, e). As previously mentioned above, both eVLP lots displayed strong signals for GP_1_ at ~140 kDa using the 6D8 antibody. However, the H3D5 antibody revealed the presence of truncated products previously seen in the test eVLP lot and the unpurified rGP material. These truncated products are highly abundant in the eVLP lot ‘E’, which returned the lowest concentration of GP_1_ by LC-HRMS quantitation with the largest ΔS value (25.5 %), whereas eVLP lot ‘A’ appears to have fewer detectable GP_1_ fragments, and returned a ΔS value of 15.8 %.

### Correlates of VLP efficacy

**Fig. 6:**
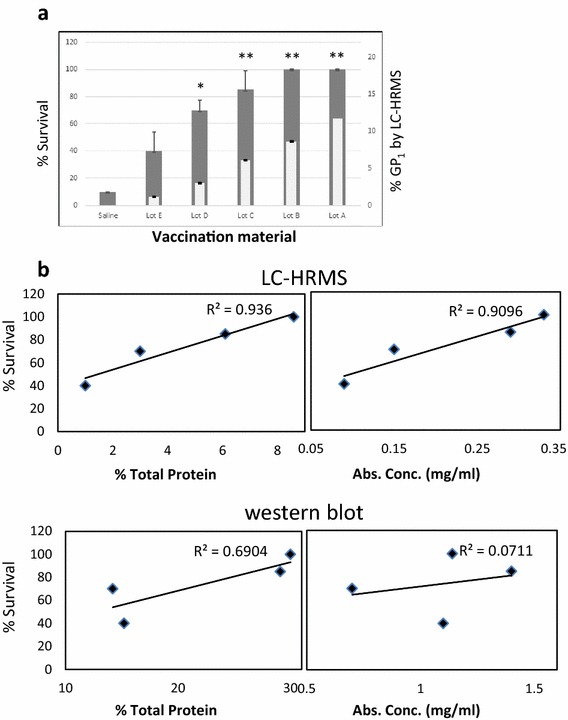
Survival data in mice for quantified eVLP lots and correlation with LC-HRMS and western blot quantitation. **a** Average percent survival (*grey bars*) after two vaccinations in mice (n = 10 for each eVLP lot) with 10 µg of GP_1_ (as calculated from 6D8 western blot results) of the indicated lot. Vaccinations were 3 weeks apart, with 4 weeks between the final vaccination and the EBOV challenge. The percent GP_1_ to total protein concentration (*right axis*) obtained using LC-HRMS is represented by the *white bars*. Survival in all vaccinated groups was significant (p < 0.005) when compared to saline controls. Fisher’s Exact test was used to compare survival between the group vaccinated with eVLP lot ‘E’ and the other lots (*indicates p < 0.01, **indicates p < 0.001). **b** LC-HRMS and western blot values for GP_1_ content in each eVLP lot tested (based on % total protein or mg/mL) were plotted against percent survival in mice after EBOV challenge (note: since Lots A and B both gave 100 % the lower lot B value was used resulting in a total of 4 data points). The strongest linear correlation (R^2^ = 0.936) was obtained with the LC-HRMS GP_1_ values based on % total protein followed by the LC-HRMS values for GP_1_ in mg/mL (R^2^ = 0.9096)

In the hopes of using immune correlates as another measure of eVLP quality, the western blot and LC-HRMS quantitation results were compared to survival data in mice for each of these eVLP vaccine preparations. Each lot of eVLP was used to immunize mice (n = 20) which were then challenged with a murine adapted EBOV. For each vaccination dose, volumes of eVLP were used which were surmised to contain 10 µg of GP (as determined from the western blot quantitation performed by the contractor). As shown in Fig. 6a, lot ‘E’ exhibited the lowest average survival rate after Ebola challenge (40 %), and animals vaccinated with the lots ‘A’ and ‘B’ exhibited 100 % survival. Lot ‘E’ contained the lowest calculated GP_1_ concentration as determined by LC-HRMS whereas the lot ‘A’ contained the highest. The difference in survival between vaccination with lots ‘A’ and ‘E’ was significant (p = 0.001). As shown in Fig. 6b, if we plot the percent survival versus the GP_1_ LC-HRMS quantitation in each eVLP lot, expressed as percent total protein, a strong positive linear correlation is observed (R^2^ = 0.936). A weaker correlation is observed if the absolute quantitation values for GP_1_ (expressed as mg/mL) obtained by LC-HRMS are plotted versus survival (p = 0.9096). In contrast western blot quantitation values (GP_1_ as a percentage of total protein) did not display strong linear correlation with percent survival (R^2^ = 0.6904) and there was essentially no correlation observed between the western blot GP_1_ concentrations (expressed as mg/mL) and survival (R^2^ = 0.0711).

The western blot quantitation of lot ‘E’ returned a value of 1.1 mg/mL of GP_1_ (see Table 4). Therefore a 10 µg GP_1_ dose would require 9.1 µL of the eVLP preparation for vaccination. However, based on the LC-HRMS quantitation, we can retroactively estimate that the animals were given only 0.82 µg of the 10 µg dose desired, which was adequate to protect only 4/10 animals vaccinated. Conversely, the western blot concentration for lot ‘A’ (1 mg/mL) is also higher than the LC-HRMS quantitation (0.55 mg/mL), and the 10 µL dose thought to contain 10 µg of GP_1_ actually contained 5.5 µg which was adequate to protect 100 % of the vaccinated animals after Ebola challenge. Therefore the observed differences in eVLP efficacy between eVLP lots ‘A’ and ‘E’ are due to vastly different concentrations of antigenic GP_1_. From the LC-HRMS quantitation of lot ‘B’, which also provided 100 % survival, we can determine that a vaccine dose (based on the western blot quantitation) of 10 ug GP_1_ would actually contain 2.89 µg which appears to be the minimal vaccination dose required to confer 100 % survival in mice after Ebola challenge.

## Discussion

Provided that technical pitfalls such as incomplete protein extraction, incomplete proteolysis or protein side-chain modifications are appropriately controlled and considered, protein quantitation by MS using an AQUA strategy employing stable isotope labelled peptides can be robust, accurate and reproducible, while achieving low limits of detection [28–30]. Ideally, target peptides should be well separated on a protein of interest to ensure that the entire protein is sufficiently denatured and digested prior to quantitation. Additionally, potential sites of post-translational modifications or residues susceptible to artefactual modifications should be avoided. In reality peptide selection is an empirical process that balances ideal characteristics with practical limitations. For example, large proteins yield more potential target peptides than small proteins, and sequence features often limit the number of possible suitable target peptides. As we discovered with the development of a quantitation assay for Ebola GP_1_, a protein of interest may have significant sequence homology with other protein species in a complex mixture, making it difficult to adhere to the peptide selection criteria described above. The quantitation of the Ebola GP_1_ in eVLP preparations was a unique challenge due to the fact that during eVLP production and purification, truncated forms of the protein are produced and retained throughout the post-production processing. This prevented the use of target peptides located in the first ~200 amino acids of the GP_1_ sequence. Additionally very few suitable target peptides were available toward the C-terminus of the protein due to the high frequency of glycosylation sites and high hydrophobicity. Therefore we have chosen a non-traditional strategy of full scan LC-HRMS quantitation in which 4 target peptides representing 2 fully tryptic and 2 missed cleavage peptides were employed. Previous studies have shown that the use of isotopically labelled target peptides with missed cleavages in protein quantitation results in no significant differences in precision, accuracy, specificity, and sensitivity compared with the use of fully tryptic peptides [31]. In addition, to further validate the assay, a purified GP_1_ standard was generated to provide quality control (for trypsin digestion parameters) and assay validation.

The average percent accuracy of our method based on quantitation of the rGP1 standard by AAA was 82.5 %. While the HPLC fractionation we performed resulted in a significant enrichment of GP_1_ from GP_2_ and truncated products of GP, contaminating protein species may still be contributing to the final concentration based on AAA. Indeed, the H3D5 western blot of the purified rGP_1_ revealed immune-reactive species of lower molecular weight, which may be the source of this overestimation, ultimately contributing to the apparent reduced accuracy of the LC-HRMS method.

The presence of truncated GP products in the eVLP preparations is the likely source of variation between the quantitation of GP_1_ with the two standard peptide sets. This hypothesis is supported by the data obtained during the rGP_1_ standard assay development and testing. The LC-HRMS quantitation of purified rGP_1_ resulted in an average ΔS of 5.1 % as compared to the eVLP method validation and reproducibility trials, which showed an average ΔS of 17.2 %, and the unpurified rGP material which routinely exhibited ΔS values of 12–13 % (data not shown). Furthermore, we have never observed a higher quantitation result for GP_1_ from peptide Set 1 (SEE/IRSEE) located in the middle of the molecule as compared to results obtained with peptide Set 2 (SVG/SVG.R) located near the N-terminus, in any of the eVLP preparations. Therefore, N-terminal sequence fragments containing the SVG region of the protein are indeed part of the GP protein compliment of eVLP and the moderate variability shown in our data is the result of experimental variation only.

Targeted MS approaches, in particular selected reaction monitoring (SRM) employing triple quadrupole mass spectrometers, have become the standard technique for quantitatively analyzing tens to hundreds of peptides and/or small molecules across a large number of samples. Unfortunately, the relatively low resolution of precursor *m*/*z* selection parameters can allow interference from nominally isobaric background contaminants especially when interrogating complex mixtures. Additionally, because only a single parent/product ion pair is monitored and no mass spectra are acquired, SRM experiments provide little to no qualitative information. Newer instrumentation platforms and configurations have facilitated the use of high-resolution accurate MS for quantitative analysis. This approach is often referred to as LC-HRMS and provides both qualitative and quantitative information during analysis by providing full-scan accurate mass data for the entire chromatographic run [32, 33]. The comprehensive detailed data obtained for each sample after LC-HRMS analysis was crucial for the development of a successful quantitation strategy for Ebola GP_1_ in eVLP preparations. For example, common modifications such as deamidation can often cause isotopic interferences, particularly when SRM-based methods are employed using low-resolution MS [32]. Conversely, full-scan HRMS data allowed the unequivocal confirmation of deamidated endogenous target peptide species which improved the accuracy of our quantitation method. Furthermore, high resolution MS/MS survey scans proved to be essential for the optimization and assessment of digestion efficiency. As revealed in this work, the detailed data provided by LC-HRMS was essential to overcome the bioanalytical challenge of GP_1_ quantitation in eVLPs, and allowed us to address potential issues prior to development of a future more streamlined quantitation scheme.

This study also highlights the superiority of mass spectrometry methods such as SRM and LC-HRMS for protein quantitation and characterization over western blotting and other immuno-affinity methods, which has been the topic of discussion in recent review articles [34, 35]. A western blot assay depends on the specificity of a single antibody, and quantitative information often relies on a protein standard that may be poorly characterized, especially if evaluation is also based on reactivity to a single antibody. This can lead to quantitative inconsistencies such as those which we observed in the GP_1_ western blot quantitation performed after eVLP production. However, there is definite value in validation by orthogonal immune-affinity approaches, and the use of the H3D5 antibody allowed us to confirm the presence of shorter versions of the GP_1_ protein in eVLP preparations and unpurified recombinant GP material.

The use of crudely purified rGP material in the western blot quantitation unintentionally led to an overestimation of the final GP_1_ concentration in eVLP, since the total protein concentration for the unpurified recombinant GP also included GP_2_ as well as truncated protein species. Data obtained from the eVLP mouse vaccination study revealed that the amount of GP_1_ in each eVLP lot as determined by LC-HRMS, unlike the quantitative western blot, correlated with survival after Ebola challenge. The highest observed correlation with animal survival was obtained using the percent of GP_1_ in relation to the total protein in the VLP. This would suggest that the “density” of GP_1_ in relation to other proteins (both viral and host derived) present in the eVLP particle is directly related to the efficacy of that particular VLP preparation.

The impact of the truncated products on eVLP quality or suitability for vaccination has not been determined. However, the LC-HRMS data revealed that the eVLP lots which exhibited the lowest percent survival (lots ‘D’ and ‘E’) also contained the lowest amount of GP_1_ and the highest ΔS values (indicating an abundance of truncated GP_1_ products). These truncated products may be the result of a frame-shift anomaly, ribosomal slippage or simply general protein degradation. Due to the fact that the secreted form of GP (sGP) is produced in greater abundance during a natural infection than GP_1,2_, and since the proteins share a common N-terminus, it has been speculated that sGP functions as a decoy molecule for EBOV-specific neutralizing and non-neutralizing antibodies [36]. Additionally, recent studies have shown that sGP actively subverts the host immune response to induce cross-reactivity with epitopes it shares with membrane-bound GP_1,2_ [37]. Therefore, truncated versions of the GP_1_ protein may indeed compromise the quality and effectiveness of eVLP vaccines, and lots exhibiting a high level of these fragments should not be used for vaccination studies.

## Conclusions

A LC-HRMS approach resulted in the successful quantitation of GP_1_ in eVLP vaccine preparations. The use of this newly developed assay will allow us to monitor variability based on GP_1_ content, providing quality control information to further optimize and refine the eVLP production process for vaccine studies. Finally, using this quantitative LC-HRMS approach, the total amount of GP_1_ necessary to confer protection can be accurately determined; a crucial factor in successful vaccine development.

## Additional files

10.1186/s12014-016-9119-8 Detection of variable missed-cleavage peptide species in the 2 quantitation peptide sets.
10.1186/s12014-016-9119-8 XIC profiles of each peptide charge state used in the quantitation showing the retention time alignment at 4 ppm. Data was taken from eVLP lot ‘B’ dilution ‘2’ replicate. The 4 profiles from each peptide are ordered from AQUA 2+ (H 2+), analyte 2+ (L 2+), AQUA 3+ (H 3+) and analyte 3+ (L 3+). A) Represents the SVG and SVGR peptide Set 2, B) is the SEE and IRSEE peptide Set 1 and C) shows the XIC profiles of the deamidated SVG and SVGR analyte peptides. XIC values were acquired with at least 7 data points sampled across the elution profile.
10.1186/s12014-016-9119-8 The binary behavior of the 2 asparagines (N) within the SVG peptide. In order to assess whether the AQUA SVG peptide counts would show a similar response to the deamidated analyte SVG peptide a 40 pmol aliquot of the AQUA peptide SVG standard was incubated at 55°C for 2.5 days at pH 8.1 resulting in complete deamidation likely at asparagine 9. Target m/z values were 969.4969 (A), 646.6684 (B), 969.0069 (C), 646.3404 (D), 969.9909 (E**)** and 646.9964 (F). The most abundant species by far is the singly deamidated 2+ ion followed by the singly deamidated 3+ ion. The doubly deamidated ions comprise 5% of the total counts. *****Note the complete absence of signal in the non-deamidated mass ranges.
10.1186/s12014-016-9119-8 LC-MS/MS protein identification of coomassie blue stained bands. (A) SDS-PAGE analysis using reducing conditions derived from a 5 µg aliquot of the eVLP lot ‘B’. The 10 highest intensity bands from a coomassie stained 4-12% NuPAGE Novex Bis-Tris gel were excised and digested with Trypsin/Lys-C. Each sample was analyzed on a 60 minute LC-MS/MS survey MS run and searched with MASCOT (v. 2.4) against the SProt (2014_02) database with human and *Ebola zaire* taxonomy specified. Locations of the 5 most prominent peptides are indicated as well as the total number of GP_1_ peptides observed for each sample (GP_1_ peptide counts). A minimum Expect score of 0.005 with an FDR of 1% was used for peptide validation. No N-terminal peptides downstream of AA 192 were observed in gel bands 7 thru 10. These data indicate that while the majority of the GP_1_ protein appears to be full-length, a significant number of N-terminal GP_1_ fragments are present. (B) An identical sample was analyzed via western blot using the H3D5 antibody showing immune-reactive bands of lower molecular weight.
