# Supplementary material for: Development of a liquid chromatography high resolution mass spectrometry method for the quantitation of viral envelope glycoprotein in Ebola virus-like particle vaccine preparations
Source: Clin Proteomics. 2016 Sep 5;13(1):18. doi: 10.1186/s12014-016-9119-8 (PMC5011338; doi:10.1186/s12014-016-9119-8)
Supplement: Supplementary file 1 — 10.1186/s12014-016-9119-8 Detection of variable missed-cleavage peptide species in the 2 quantitation peptide sets. [file 12014_2016_9119_MOESM1_ESM.pdf]

Supplemental Table 1. Detection of variable missed-cleavage peptide species in the 2 quantitation peptide sets

Sequence	Total Area	% Area
SEELSFTAVSNR	1113002	90.70%
**IR**SEELSFTAVSNR	100520	8.20%
**KIR**SEELSFTAVSNR	13555	1.10%
**IR**SEELSFTAVSNR**AK**	ND	NA
**KIR**SEELSFTAVSNR**AK**	ND	NA
**NLTRKIR**SEELSFTAVSNR	ND	NA
**NLTRKIR**SEELSFTAVSNR**AK**	ND	NA
Sequence	Total Area	% Area
SVGLNLEGNGVATDVPSATK	923739	68.70%
SVGLNLEGNGVATDVPSATK**R**	420694	31.30%
SVGLNLEGNGVATDVPSATK**RWGFR**	ND	NA
**LSSTNQLR**SVGLNLEGNGVATDVPSATK	ND	NA
**LSSTNQLR**SVGLNLEGNGVATDVPSATK**R**	ND	NA

ND = Not Detected
