# Supplementary figures and images for: Development of a liquid chromatography high resolution mass spectrometry method for the quantitation of viral envelope glycoprotein in Ebola virus-like particle vaccine preparations
Source: Clin Proteomics. 2016 Sep 5;13(1):18. doi: 10.1186/s12014-016-9119-8 (PMC5011338; doi:10.1186/s12014-016-9119-8)

Supplemental Figure 1

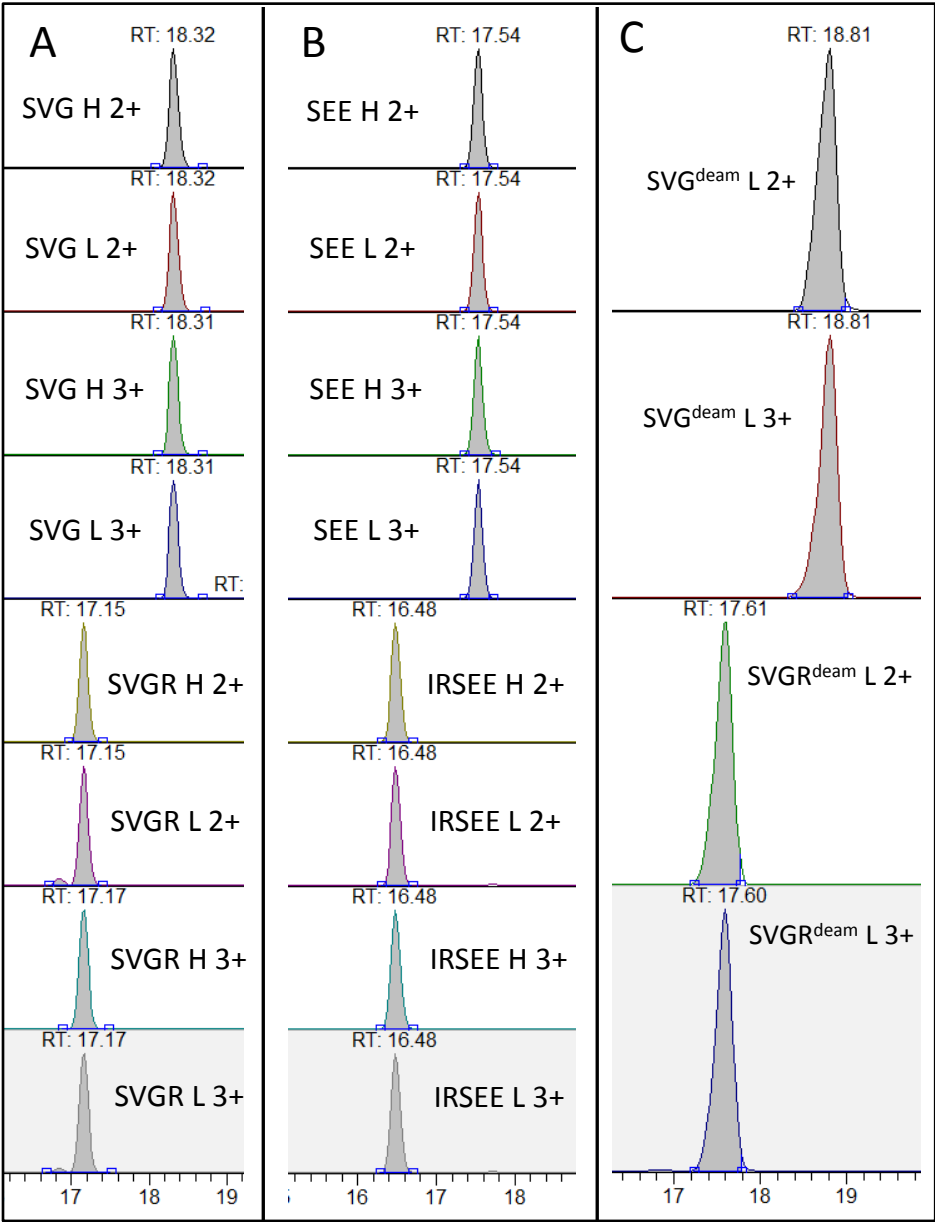

Supplement: Supplementary file 2 — 10.1186/s12014-016-9119-8 XIC profiles of each peptide charge state used in the quantitation showing the retention time alignment at 4 ppm. Data was taken from eVLP lot ‘B’ dilution ‘2’ replicate. The 4 profiles from each peptide are ordered from AQUA 2+ (H 2+), analyte 2+ (L 2+), AQUA 3+ (H 3+) and analyte 3+ (L 3+). A) Represents the SVG and SVGR peptide Set 2, B) is the SEE and IRSEE peptide Set 1 and C) shows the XIC profiles of the deamidated SVG and SVGR analyte peptides. XIC values were acquired with at least 7 data points sampled across the elution profile. [file 12014_2016_9119_MOESM2_ESM.pdf]

Supplemental Figure 2

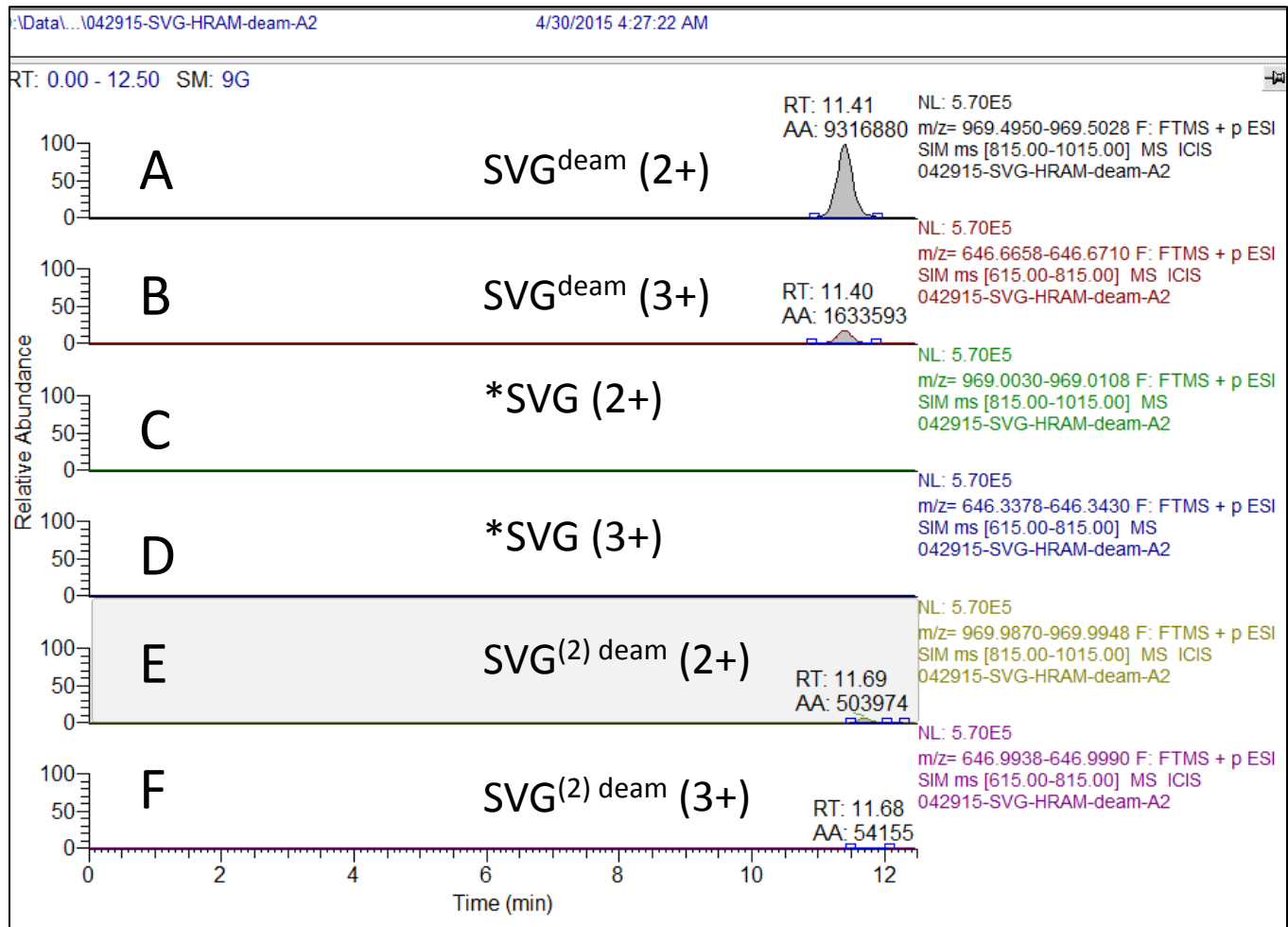

Supplement: Supplementary file 3 — 10.1186/s12014-016-9119-8 The binary behavior of the 2 asparagines (N) within the SVG peptide. In order to assess whether the AQUA SVG peptide counts would show a similar response to the deamidated analyte SVG peptide a 40 pmol aliquot of the AQUA peptide SVG standard was incubated at 55°C for 2.5 days at pH 8.1 resulting in complete deamidation likely at asparagine 9. Target m/z values were 969.4969 (A), 646.6684 (B), 969.0069 (C), 646.3404 (D), 969.9909 (E) and 646.9964 (F). The most abundant species by far is the singly deamidated 2+ ion followed by the singly deamidated 3+ ion. The doubly deamidated ions comprise 5% of the total counts. *Note the complete absence of signal in the non-deamidated mass ranges. [file 12014_2016_9119_MOESM3_ESM.pdf]

Supplemental Figure 3

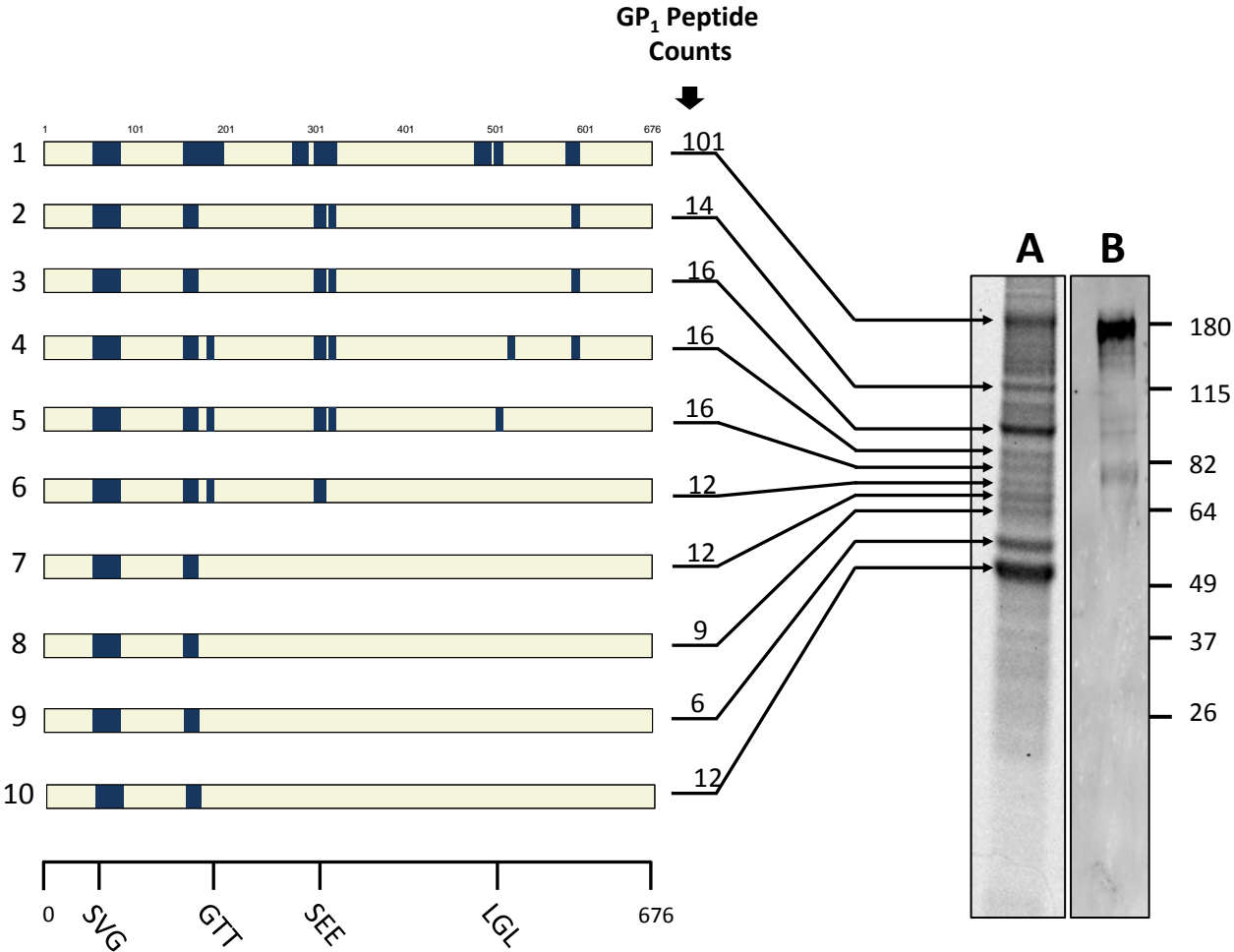

Supplement: Supplementary file 4 — 10.1186/s12014-016-9119-8 LC-MS/MS protein identification of coomassie blue stained bands. (A) SDS-PAGE analysis using reducing conditions derived from a 5 µg aliquot of the eVLP lot ‘B’. The 10 highest intensity bands from a coomassie stained 4-12% NuPAGE Novex Bis-Tris gel were excised and digested with Trypsin/Lys-C. Each sample was analyzed on a 60 minute LC-MS/MS survey MS run and searched with MASCOT (v. 2.4) against the SProt (2014_02) database with human and Ebola zaire taxonomy specified. Locations of the 5 most prominent peptides are indicated as well as the total number of GP1 peptides observed for each sample (GP1 peptide counts). A minimum Expect score of 0.005 with an FDR of 1% was used for peptide validation. No N-terminal peptides downstream of AA 192 were observed in gel bands 7 thru 10. These data indicate that while the majority of the GP1 protein appears to be full-length, a significant number of N-terminal GP1 fragments are present. (B) An identical sample was analyzed via western blot using the H3D5 antibody showing immune-reactive bands of lower molecular weight. [file 12014_2016_9119_MOESM4_ESM.pdf]
